# Caveolin-1 - A Novel Interacting Partner of Organic Cation/Carnitine Transporter (Octn2): Effect of Protein Kinase C on This Interaction in Rat Astrocytes

**DOI:** 10.1371/journal.pone.0082105

**Published:** 2013-12-13

**Authors:** Magdalena Czeredys, Łukasz Samluk, Katarzyna Michalec, Karolina Tułodziecka, Krzysztof Skowronek, Katarzyna A. Nałęcz

**Affiliations:** 1 Department of Molecular and Cellular Neurobiology, Nencki Institute of Experimental Biology, Warsaw, Poland; 2 Laboratory of Bioinformatics and Protein Engineering, International Institute of Molecular and Cell Biology, Warsaw, Poland; 3 Department of Biochemistry, Nencki Institute of Experimental Biology, Warsaw, Poland; University of Cambridge, United Kingdom

## Abstract

OCTN2 - the Organic Cation Transporter Novel family member 2 (SLC22A5) is known to be a xenobiotic/drug transporter. It transports as well carnitine - a compound necessary for oxidation of fatty acids and mutations of its gene cause primary carnitine deficiency. Octn2 regulation by protein kinase C (PKC) was studied in rat astrocytes - cells in which β-oxidation takes place in the brain. Activation of PKC with phorbol ester stimulated L-carnitine transport and increased cell surface presence of the transporter, although no PKC-specific phosphorylation of Octn2 could be detected. PKC activation resulted in an augmented Octn2 presence in cholesterol/sphingolipid-rich microdomains of plasma membrane (rafts) and increased co-precipitation of Octn2 with raft-proteins, caveolin-1 and flotillin-1. Deletion of potential caveolin-1 binding motifs pointed to amino acids 14–22 and 447–454 as the caveolin-1 binding sites within Octn2 sequence. A direct interaction of Octn2 with caveolin-1 in astrocytes upon PKC activation was detected by proximity ligation assay, while such an interaction was excluded in case of flotillin-1. Functioning of a multi-protein complex regulated by PKC has been postulated in rOctn2 trafficking to the cell surface, a process which could be important both under physiological conditions, when carnitine facilitates fatty acids catabolism and controls free Coenzyme A pool as well as in pathology, when transport of several drugs can induce secondary carnitine deficiency.

## Introduction

Several solute transporters are important for proper functioning of astrocytes, moreover, their activity is a necessary prerequisite of a close cooperation in the brain between astrocytes and neurons, just to mention maintenance of neurotransmitters pool. Organic cation/carnitine transporter OCTN2 [1]–[3], coded by *SLC22A5* gene, belongs to a superfamily of organic ion transporters, specific towards organic anions (OATs), urate (URAT) and organic cations (OCTs and OCTNs) for review see, [4]. The OCTN family comprises 3 known members, out of which OCTN1 has been reported to be specific for ergothioneine [5], while OCTN2 and OCTN3 are high affinity carnitine transporters [2], [3]. OCTN3 has been postulated to function as a peroxisomal carnitine transporter [6], [7]. OCTN2, in a Na^+^-independent way, transports a broad spectrum of organic cations, including xenobiotics/drugs as substrates [8]–[10]. It transports as well carnitine, but in a Na^+^-dependent way [1], [2] and mutations in *SLC22A5* gene can cause systemic carnitine deficiency, classified as an inherited disease OMIM212149 [11]. OCTN2 is ubiquitous in the peripheral tissues and it was found to be present in the brain and in cultured astrocytes [6], [12]–[14].

The *octn2* was shown to be up-regulated in peripheral tissues by peroxisome proliferators activator receptor α (PPARα), what correlated with an increased expression of genes coding several enzymes involved in β-oxidation of fatty acids [15]. The observed up-regulation of *octn2* confirms an important physiological role of carnitine in transfer of acyl moieties in form of carnitine esters through the inner mitochondrial membrane. However, such an up-regulation of *octn2* by PPARα was not observed in astrocytes [6]. It has to be noted that, although astrocytes are the main brain cells capable to perform β-oxidation, this process is not a prevailing energy supply in the brain [16]. Anyhow, astrocytes, fulfill an important physiological role in other steps of brain lipids metabolism, just to mention synthesis of cholesterol [17], [18], as well as elongation and desaturation reactions of ϖ-6 and ϖ-3 essential fatty acids [19]. Thus carnitine can play an important role in transport of these lipids and their precursors through intracellular membranes.

OCTN2 can be also regulated post-transcriptionally, its mRNA was shown to be stabilized in endoplasmic reticulum by cartregulin [20]. OCTN2 function was shown as well to be regulated post-translationally by interaction with PDZ proteins - PDZK1 and PDZK2 [21], [22], although the precise mechanism leading to increased activity has not been established [10].

Many plasma membrane transporters are regulated post-translationally by phosphorylation. Analysis of OCTN2 sequence reveals presence of 6 potential protein kinase C (PKC) phosphorylation sites [1], anyhow, transporter phosphorylation has not been demonstrated. There are several reports on regulation by PKC of amino acid and neurotransmitter transporters, although the physiological effects could have been opposite. PKC activation resulted in augmented activity and an increased delivery to plasma membrane of glutamate transporters [23], while Na^+^/Cl^–^dependent neurotransmitter transporters were reported to undergo internalization upon PKC stimulation [24]–[27].

Several transporting proteins have been reported to be localized at least partially in cholesterol/sphingolipid-rich microdomains - so-called rafts [28]–[31]. Some transporters have been shown to directly interact with proteins present in cholesterol/sphingolipid-rich microdomains, as caveolin-1 [32]. In astrocytes internalization from rafts was proposed in case of an amino acid transporter ATB^0,+^ [33], a protein transporting carnitine with a low affinity [34].

Since there is no information on trafficking regulation by PKC of transporters coded by *SLC22* gene superfamily, the present work was focused on the effect of this kinase on Octn2 in astrocytes, in particular on the phosphorylation status of the transporter, its activity, localization in membrane microdomains, as well as a possible interaction with raft proteins. Apart from lack of a direct Octn2 phosphorylation by PKC in rat astrocytes, activation of this kinase was correlated with increased carnitine transport and augmented Octn2 presence in plasma membrane, in particular in rafts. A direct interaction with caveolin-1 and amino acid sequence fragments responsible for this binding have been established.

## Materials and Methods

### Materials

L-[methyl-^3^H]carnitine and inulin[^14^C]carboxylic acid were from Amersham (Buckinghamshire, UK). Polyclonal antibody against rOctn2 peptide 537–553 [35] and polyclonal antibody against ATB^0,+^ [36] were raised and delivered by GenScript Corporation (Piscataway, NJ, USA). Monoclonal anti-flotillin-1 antibody was from BD Transduction Laboratories (San Jose, CA, USA). Anti-caveolin-1 monoclonal (7C8) and polyclonal (N-20) antibodies were from Novus Biologicals (Littleton, CO, USA) and Santa Cruz Biotechnology (Santa Cruz, CA, USA), respectively. Monoclonal antibodies against phosphoserine (clones PSER-7F12 and PSER-4A9) were from Enzo Life Sciences (Biomibo, Poland). LR WHITE was from Polyscience Europe GmbH (Eppelheim, Germany). Protein A Sepharose™ CL-4B was from GE Healthcare Bio-Sciences (Uppsala, Sweden). EZ-Link® Sulfo-NHS-LC-Biotin [Sulfosuccinimidyl-6-(biotinamido)hexanoate] and Pierce® Avidin Agarose Resin were from Pierce (Rockford, IL, USA). Proximity ligation assay kit was from OLINK Bioscience (Uppsala, Sweden). Lipofectamine™ 2000 and fetal bovine serum were from Invitrogen (Grand Island, NY, USA). Nonidet P40 and phosphatase inhibitor cocktail PhosSTOP were from Roche (Mannheim, Germany). Ampholine (Bio-Lyte, pH 3–10) and immobilized pH gradient (3–10) strips for first dimension protein separation were from BioRad (Warsaw, Poland). Phusion High-Fidelity DNA, T4 Polynucleotide Kinase and T4 DNA Ligase were from Thermo Scientific (Gdańsk, Poland). Monoclonal anti-phosphoserine antibody (clone PSR-45), monoclonal anti-FLAG M2 antibody, donkey anti-rabbit antibodies conjugated with 15 nm colloidal gold particles and donkey anti-mouse antibodies conjugated with 10 nm colloidal gold particles, anti-FLAG M2 affinity agarose gel, succinyl-concanavalin A-fluorescein isothiocyanate (FITC) labeled, Taq polymerase, Enhanced Avian HS RT-PCR kit and all other reagents were from Sigma (Poznań, Poland).

### Plasmid Construction

RNA was isolated from rat kidney with TRIzol, subjected to reverse transcription PCR with Enhanced Avian HS RT-PCR kit (Sigma). cDNA coding rOctn2 (accession No. NCBI AF110416) was amplified with the primers 5′-TAAAGCTTATGCGGGACTACGAC-3′ and 5′-TAGGATCCGAAGGCTGTGCTCTTTAG-3′ (introduced recognition sites for *Hin*dIII and *Bam*HI respectively are underlined) with annealing temperature of 58.5°C. Product of the reverse transcription was reamplified with the same primers and Taq polymerase. The final product was cloned as *Hin*dIII-*Bam*HI fragment in pBluescript II KS (+) vector (pBluescript II KS (+)/OCTN2). In order to delete potential caveolin-1 binding motif corresponding to amino acids 14–22, the pBluescript II KS (+)/OCTN2 was amplified with the primers 5′-TTCGCCCAGGAAGGCGGTC-3′ and 5′-TTCCTGCTCAGCGCCAGC-3′ (introducing recognition sites for *Eco*RI upon ligation) using Phusion polymerase without annealing step. 5′ ends of PCR product were phosphorylated with T4 Polynucleotide Kinase, followed by circularization with T4 DNA Ligase and transformation of *E. coli* TOP10 with the ligation mixture (pBluescript II KS (+)/OCTN2-Δ14–22). The plasmids pBluescript II KS (+)/OCTN2 and pBluescript II KS (+)/OCTN2-Δ14–22 were used for deletion of the second potential caveolin-1 binding site, encompassing amino acids 447–454 of rOctn2. Plasmids pBluescript II KS (+)/OCTN2 and pBluescript II KS (+)/OCTN2-Δ14–22 were subjected to PCR with the primers 5′-CCCACTGTGGTCAGAAAC-3′ and 5′-TACCATGGAATAGGCAGAG-3′ (introducing upon ligation recognition site for *Kpn*I) and with Phusion polymerase with annealing temperature of 55°C. 5′ ends of PCR products were phosphorylated with T4 Polynucleotide Kinase, ligated with T4 DNA Ligase and the ligation mixtures were used for *E. coli* TOP10 transformation to obtain pBluescript II KS (+)/OCTN2-Δ447–454 and pBluescript II KS (+)/OCTN2-Δ14–22/Δ447–454. All the constructs were isolated and inserts were recloned in p3xFLAG-CMV14 vector as *Hin*dIII-*Bam*HI fragments. Final constructs expressed recombinant proteins with C-terminal 3xFLAG fusion and were used for transfection of HEK293 cells. All plasmid constructs were verified by DNA sequencing.

### Cell Culture and Experimental Pretreatment

As described previously [6], the astrocytes were isolated from 3-day old Wistar albino (WAG) rats. Prior to experiments rats were cared in the Animal House of the Nencki Institute of Experimental Biology (Warsaw, Poland), following the recommendation in the Guide for the Care and Use of Laboratory Animals of the National Institutes of Health. The 3-day old rats were sacrificed by decapitation following the protocol approved by the First Local Ethics Committee on Animal Experimentation (Warsaw, Poland), according to Polish Law on Experimentation on Animals implementing the European Council Directive 86/609/EEC on the protection of animals used for scientific purposes (permits numbers 1009/2009 and 260/2012). Astrocytes were isolated as described in [6]. Both, fibroblasts NIH/3T3 (ATTC®- American Type Culture Collection) and astrocytes were cultured in 10% fetal bovine serum inactivated at 56°C, 90% Dulbecco’s modified Eagle medium supplemented with 2 mM L-glutamine and gentamycin (100 µg/ml) at 37°C in a humid atmosphere of 5% CO_2_. For the experiments, astrocytes were treated either with 0.1% dimethylsulfoxide as vehicle (controls) or with 10 µM bisindolylmaleimide II (Bis II) for 5 min followed by 30 min treatment with 200 nM phorbol 12-myristate 13-acetate (PMA), or with PMA alone. Transfection of HEK293 cells (ATTC®) with either p3xFLAG-CMV14 or plasmids carrying *octn2* inserts was performed with Lipofectamine™ 2000, according to supplier protocol. The cells were cultured as given in [36].

### Flow Cytometry

HEK293 cells were washed twice with phosphate buffered saline (PBS), detached with trypsin and collected by centrifugation (170×g, 3 min). Samples (10^6^ cells) were treated with 4% paraformaldehyde for 15 min (on ice), spun down and incubated for 15 min at 20°C in PBS supplemented with 0.1% Triton X-100. The cells were collected by centrifugation, unspecific sites were blocked with 10% goat serum in PBS, followed by centrifugation and incubation with anti-FLAG monoclonal antibody (1∶1000) for 1 h at 20°C. After 2 washes with PBS the cells were incubated for 1 h with Alexa Fluor 488 labeled anti-mouse antibody (1∶1000), followed by washes with PBS. The cells were suspended in 500 µl PBS and Alexa Fluor 488 fluorescence was determined by flow cytometry on a FACS Calibur with a 488 nm green laser in a FL1 channel.

### Immunoprecipitation and Western Blot Analysis

Astrocytes were homogenized and analysed by Western blotting, as given in [33] and the membranes were probed with either anti-Octn2 antibody (1∶100) or anti-caveolin-1 (1∶400), anti-flotillin-1 (1∶500) or anti-phosphoserine (1∶100) antibodies, as given in Figure legends. For two-dimentional electrophoresis, extracted proteins were precipitated with 25% trichloroacetic acid, washed with acetone and subsequently suspended in 8 M urea, 2% 3-[(3-cholamidopropyl)dimethylammonio]-1-propanesulfonate (CHAPS), 50 mM dithiothreitol (DTT), 0.2% ampholine, transferred onto polyacrylamide strips (pH 3–10), incubated for 1 h at 20°C, covered with paraffin oil and incubated for 16 h at 20°C. Isoelectrofocusing was performed for 20 h in Protean IEF Cell (BioRad) according to supplier protocol. The strips were washed in 6 M urea, 2% sodium dodecyl sulfate (SDS), 20% glycerol, 2% DTT, 375 mM Tris-Cl, pH 8.8, followed by washing with the same buffer in which DTT was replaced by 2% iodoacetamide. The strips were transferred on 10% polyacrylamide gels with 5% stacking gels and subjected to electrophoresis and Western blot analysis with antibodies indicated in the Figure legend. For immunoprecipitation with Protein A Sepharose™ CL-4B astrocyte homogenates were subjected to preclearance, followed by immunoprecipitation with antibodies (4 µg) against indicated proteins [33]. HEK293 cells transfected with p3xFLAG-CMV14 alone or with plasmids carrying *octn2* inserts were lysed in 150 mM NaCl, 50 mM Tris, pH 7.4 supplemented with 1% Nonidet P40, protease inhibitor cocktail and phosphatase inhibitors and after protein estimation subjected either to immunoblotting or to immunoprecipitation with anti-FLAG M2 affinity agarose gel. Elution was performed with 3xFLAG® PEPTIDE (7.5 µg). The blots were analysed with antibodies indicated in Figures legends. The quantitative analysis of the bands intensity was performed with the INGENIUS apparatus (Syngen Biotech).

### Biotinylation of Cell Surface Protein

After experimental treatment astrocytes were washed with ice-cold PBS containing 0.1 mM CaCl_2_ and 1 mM MgCl_2_ (PBS/Ca/Mg) and incubated in the same solution supplemented with 1 mg/ml EZ-Link® Sulfo-NHS-LC-Biotin for 30 min at 4°C. The free sulfo-NHS-biotin was removed by incubation with 100 mM glycine in PBS/Ca/Mg for 15 min at 4°C with shaking and two washes with ice-cold PBS/Ca/Mg. The cells were collected and homogenized in RIPA buffer (150 mM NaCl, 10 mM EDTA, 1% Triton X-100, 0.5% sodium deoxycholate, 0.1% SDS, 50 mM Tris, pH 7.4) supplemented with proteases inhibitors, followed by centrifugation at 20,000×g for 15 min at 4°C. The supernatants were collected and, after estimation of protein content, samples containing equal amounts of protein (700 µg) were mixed with 100 µl of Pierce® Avidin Agarose Resin and incubated overnight at 4°C. Biotinylated proteins were eluted with sample buffer and subjected to electrophoretic separation and Western blot analysis.

### Immunocytochemistry

HEK293 cells overexpressing rat Octn2 were washed three times with PBS and fixed with 4% paraformaldehyde at 4°C. The non-permeabilized and permeabilized cells were analysed with FITC-conjugated-concanavalin A (100 µg/ml) and anti-FLAG antibody (1∶1000), as described in [36].

A possibility of co-localization of two different proteins in astrocytes was analyzed by proximity ligation assay. Astrocytes were washed and fixed with 4% paraformaldehyde at 4°C. After consecutive washes they were permeabilized with 0.1% Triton X-100 in PBS followed by a blocking step with 10% goat serum. The cells were incubated with two primary antibodies (both at dilution 1∶100), one raised in rabbit, the other one in mouse, as indicated in the Figure legend. The next steps: washing, incubation with assay probes, ligation with the ligase and amplification with polymerase followed the supplier protocol. The analysis, after mounting in a medium with DAPI, was performed with the Leica SP5 confocal microscope, with excitation at 400 nm and emission at 405–490 nm for DAPI (nuclei) and excitation at 561 nm and emission at 580–650 nm for Reagents Red. Quantification of the fluorescence signal was referred to the amount of nuclei.

### Electron Microscopic Immunocytochemistry

HEK293 cells were fixed 1 h in 3% paraformaldehyde and 0.1% glutaraldehyde in PBS, then treated with 1% osmium tetroxide for 30 min, dehydrated in the ethanol gradient, and finally embedded in LR WHITE resin. The ultrathin sections were cut using LKB-NOVA ultramicrotome, mounted on the formvar-coated nickel grids and blocked for 1 h at room temperature in 1% BSA, 0.05% Tween-20 in PBS. All antibodies were diluted in 1% BSA, 0.05% Tween-20 in PBS. Monoclonal anti-FLAG antibody (1∶50) was applied on grids and left for 2.5 h at 20°C, followed by washing with PBS. Next the polyclonal rabbit anti-OCTN2 antibody (1∶20) was added for another 2.5 h (20°C), the grids were washed afterwards with PBS for 30 min and subsequently exposed for 2.5 h to donkey anti-mouse IgG conjugated with 10 nm colloidal gold particles (1∶100), washed 30 min in PBS and exposed to secondary donkey anti-rabbit IgG conjugated with 15 nm colloidal gold particles (1∶100). The grids were washed with distilled water and examination of all sections was carried out with JEM 1400 electron microscope (JEOL Co, Japan) at 80 kV.

### Isolation of Membrane Rafts

Astrocytes were homogenized in 150 mM NaCl, 2 mM EDTA, 10 mM Tris, pH 7.5 (buffer A), supplemented with 1% Triton X-100 and the protein extract (1.2 mg protein) was mixed 1∶1 with 80% sucrose in buffer A, placed at the bottom of 5 ml ultracentrifuge tubes and overlayered with equal volumes of 30% sucrose and 5% sucrose in the same buffer. The samples were centrifuged at 200,000×g for 24 h at 4°C in Beckman Coulter Optimal L-100 XP ultracentrifuge. Fractions (480 µl each) were collected from the top of each gradient, protein was precipitated with 25% trichloroacetic acid, spun down after 10 min and subjected to gel electrophoresis and immunoblotting.

### Transport Measurements

Astrocytes were washed and incubated in either 137 mM NaCl, 10 mM HEPES, pH 7.4 or in 137 mM sodium gluconate, 10 mM HEPES, pH 7.4 for experiments without chloride. The cells were covered with 3 ml of the corresponding buffer and preincubated with either PMA alone or PMA with Bis II, as indicated in the Figure legend. Transport of 5 M L-[methyl-^3^H]-carnitine (600 Ci/mol), with inulin[^14^C]carboxylic acid as a control, was measured, as described in [37]. In case of HEK293 cells transport was measured in cells transfected either with p3xFLAG-CMV14 vector alone or with p3xFLAG-CMV14/OCTN2. For estimation of carnitine transport the cells were dissolved by incubating overnight in 0.1 M NaOH, 2% Na_2_CO_3_, 1% SDS and samples were taken for radioactivity counting and protein estimation.

### Statistical Analysis

The mean ± SEM was calculated for each set of experiments and statistical analysis (GraphPad Prism program, GraphPad Software Inc., San Diego, CA, USA) was performed using one-way ANOVA with subsequent Bonferroni’s multiple comparison test. For Western blot analyses the representative blots have been shown and the number of independent experiments (n) has been indicated in Figure legends.

## Results

### Phorbol Ester Treatment Activates Carnitine Transport

**Figure 1:**
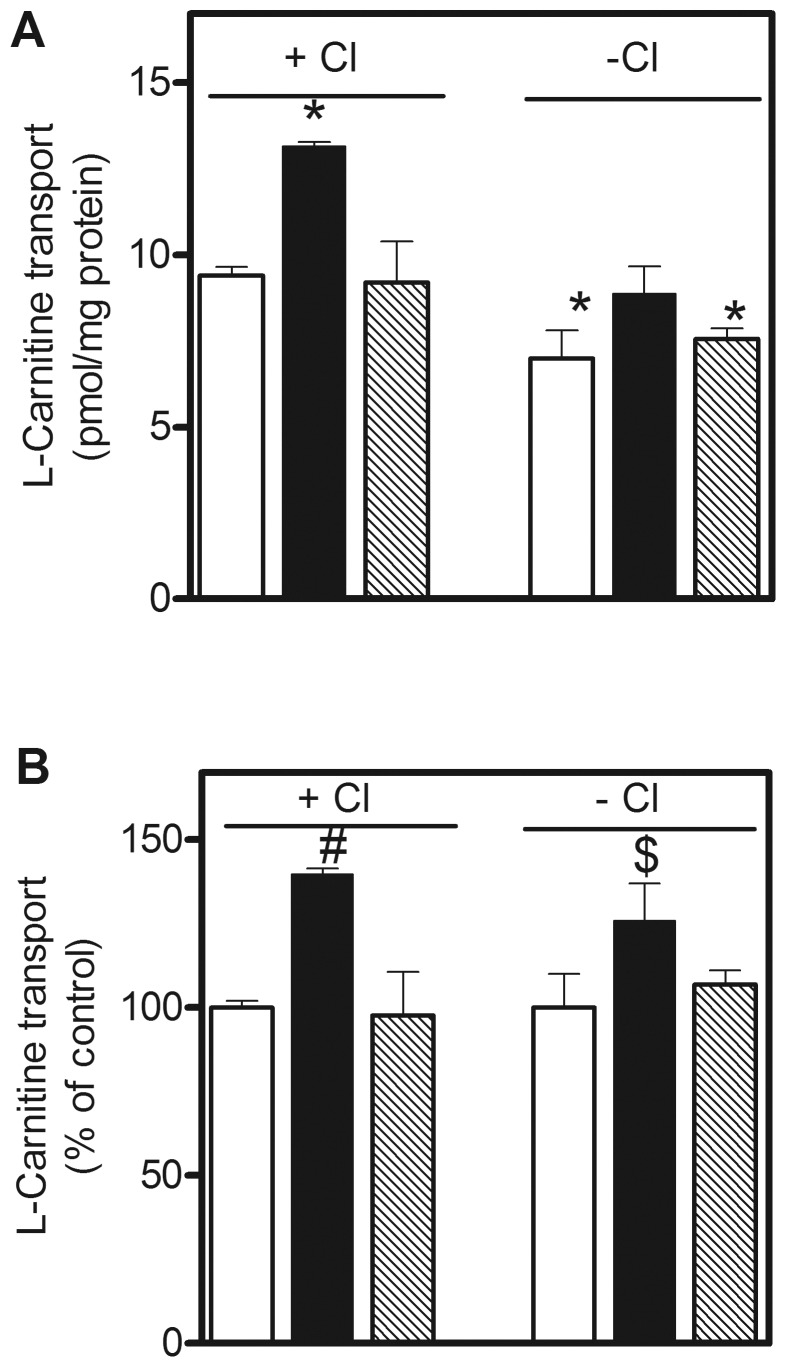
Effect of PKC activation on Octn2 activity in astrocytes. The cells were incubated either without any additions (open bars) or with PMA (filled bars) or Bis II and PMA (hatched bars) in the presence or absence of chloride ions and the initial velocity of L-carnitine transport was measured as described in Materials and Methods. (A) Results are means ± SEM (n = 6), *p<0.001 *versus* control in the presence of Cl^−^. (B) The same results shown as percent of the corresponding control ^#^p<0.001 and ^$^p<0.05 *versus* controls with and without Cl^−^, respectively.

Astrocytes transport L-carnitine (Fig. 1A), a process stimulated by 40% after pretreatment with PKC activator – PMA and reversed by Bis II. Carnitine transport in the presence of NaCl represents a sum of activity of all carnitine transporters, including a Na^+^/Cl^–^dependent amino acid/carnitine transporter ATB^0,+^ [34], known to be present in astrocytes [33], [38]. Omission of chloride, i.e. inhibition of ATB^0,+^ activity, results in a 25% decrease of carnitine transport, pointing to a substantial (75%) contribution of Octn2 in the process of carnitine accumulation in astrocytes. In the absence of Cl^−^ ions, PMA stimulated carnitine transport by 25% (Fig. 1B), indicating a possibility of Octn2 regulation by PKC.

### Phorbol Ester Increases Surface Presence of Octn2 without Transporter Phosphorylation

**Figure 2:**
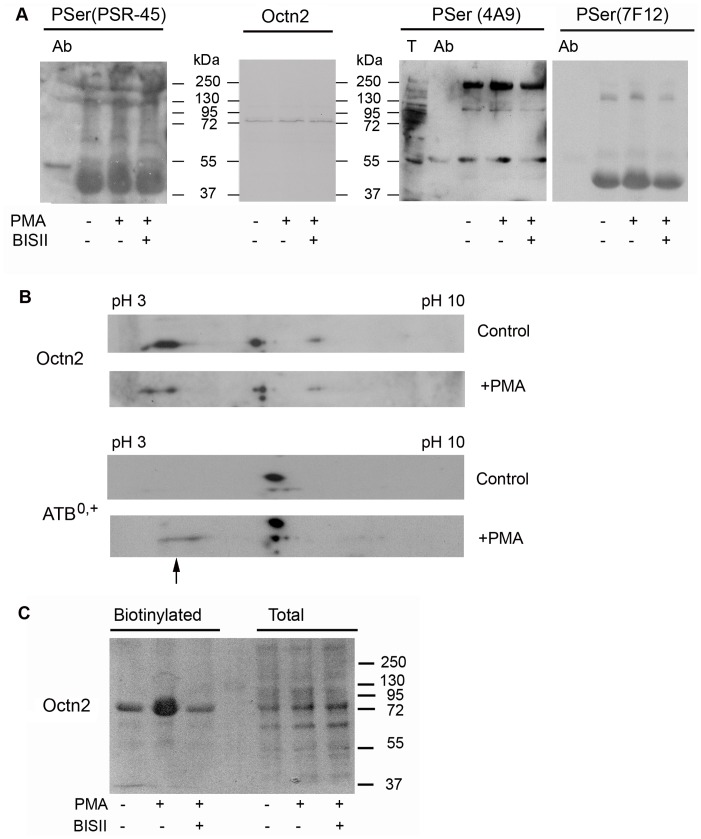
Analysis of Octn2 phosphorylation and plasma membrane presence. The cells were incubated either without any additions, or with PMA or Bis II and PMA, as described in Materials and Methods. (A) Octn2 was immunoprecipitated with anti-Octn2 antibodies and its phosphorylation status was analysed with different clones of anti-phosphoserine antibodies (PSR-45 n = 5, 4A9 n = 6, 7F12 n = 3). Ab, anti-Octn2 antibody subjected to immunoprecipitation. (B) Two-dimentional analysis of carnitine transporters Octn2 and ATB^0,+^ isolated from astrocytes (arrow indicates shift of ATB^0,+^ pI after PMA). (C) Detection of Octn2 in fraction containing biotinylated cell surface proteins (3 mg protein per analysis) and in the total extract (50 µg protein).

In order to verify, if Octn2 can be phosphorylated by PKC, the cell extracts obtained at all experimental conditions were subjected to immunoprecipitation. It has to be noted that the total amount of Octn2 was not changed (Fig. 2A). The antibody recognizing phosphorylated serine (PSR-45) detects phosphoserine moieties of the heavy immunoglobulin chain (Fig. 2A). It recognizes as well several other bands, none of them, however, migrates with the same mobility as Octn2, although it seems that PMA treatment increased phosphorylation of 3 proteins (M_r_ of 46,000, 130,000 and above 250,000). We further verified the phosphorylation status of proteins co-precipitating with Octn2 using two different clones of anti-phosphoserine antibodies recognizing phosphoserine in a vicinity of positively charged amino acids, a consensus site recognized by PKC [39]. Both clones recognized several phosphorylated bands, none of them corresponded to Octn2 itself. Interestingly, clone PSER-7F12 reacted with the same proteins of M_r_ of 130,000 and 46,000, as recognized by PSR-45. It should be emphasized that clone 4A9 was previously shown by our group to detect an increased phosphorylation of a low affinity carnitine transporter ATB^0,+^, both in astrocytes and HEK293 cells treated with PMA [33], [36]. It has to be added that neither a clone requiring proline or lysine C-terminal to phosphoserine (PSER-16B4) nor anti-phosphothreonine antibody (PTHR-4D11) gave any positive results (not shown). Lack of Octn2 detection with anti-phosphoserine antibodies could be caused as well by a small fraction of phosphorylated transporter, not detected by the applied antibodies. Therefore, we analysed the position of Octn2 transporter by two-dimentional electrophoresis. As shown in Fig. 2B, the same spots can be detected with anti-Octn2 antibody under control conditions and after PMA. On the contrary, a similar analysis of ATB^0,+^ revealed a shift of the band recognized by anti-ATB^0,+^ antibody towards more acidic pH, indicating lower pI of the protein, what could result from phosphorylation (Fig. 2B, arrow). Therefore, it seems that the observed stimulation of carnitine transport in astrocytes by PKC activator does not result from a direct phosphorylation of the transporter, but rather from augmented phosphorylation of other proteins interacting with Octn2.

We wanted to verify further, if the increase in transport activity could result from the changed surface presence of Octn2. Plasma membrane proteins were biotinylated and separated with use of avidin resin. Western blot analysis of biotinylated fraction for Octn2 presence shows its dramatic increase (by 80%) after PMA (Fig. 2C). Since a total amount of Octn2 is not changed, this observation points to existence of a large intracellular pool of the transporter and its significant movement to plasma membrane upon PKC activation.

### PMA Increases Octn2 Localization in Rafts

**Figure 3:**
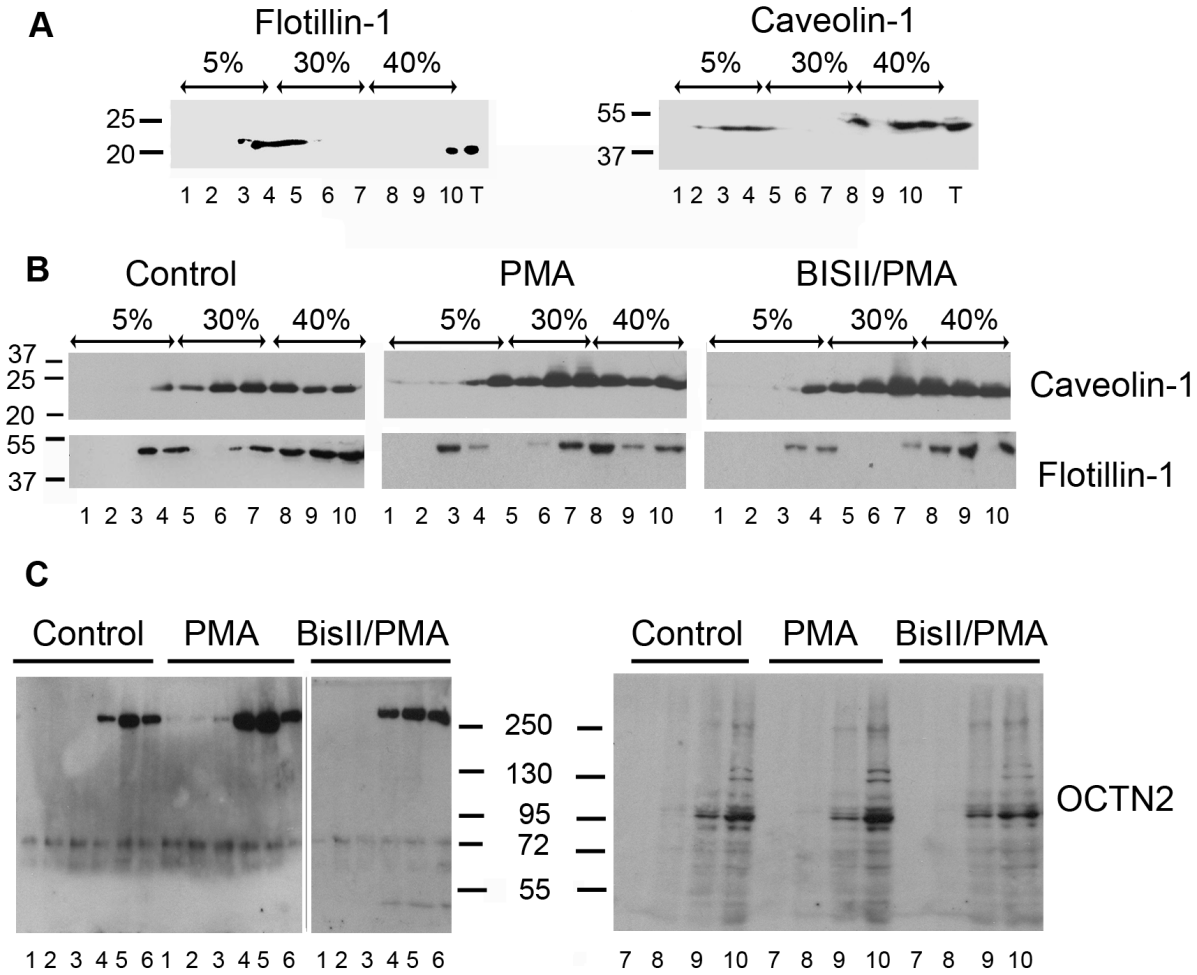
Localization of Octn2 in different membrane domains. Distribution of raft proteins: flotillin-1 and caveolin-1 in sucrose gradient fractions after solubilization with Triton X-100 of fibroblasts NIH/3T3 (A) (n = 2) and astrocytes (B) (n = 5). In case of Octn2 for analysis of upper fractions (C, left panels) 3 times more fraction volume was taken than from the bottom fractions (C, right panel) (n = 5).

In order to clarify, if an increased amount of Octn2 in plasma membrane could reflect its localization within rafts, a more detailed analysis of Octn2 presence in subdomains of plasma membrane was performed by sucrose gradient centrifugation after solubilization in the presence of Triton X-100. Two proteins, known to be present in rafts, caveolin-1 [40] and flotillin-1 (reggie-2) [41] were taken as reference. Although they were separated in floating fractions of sucrose gradient when the analysis was performed with 3T3 cell (Fig. 3A), in astrocytes, as already observed before [33], flotillin-1 and caveolin-1 are detected in lower fractions (Fig. 3B). Such localization of rafts proteins in astrocytes results most probably from the presence of cytosolic lipid-protein particles associated with microtubules detected previously in these cells [42]. Anyhow, the amount of both proteins was observed to increase in floating fractions (at the border of 5% sucrose) after PMA treatment. It was impossible to perform a similar analysis for Octn2 at the same gel and blot, since the majority of the transporter (70–80%) was found in fractions containing higher sucrose concentrations. Therefore the upper and lower fractions were analysed separately with a 3-fold higher volume of floating fractions taken for Octn2 detection and, in order to verify influence of PMA on Octn2 localization in detergent-resistant domains, the upper fractions from control and PMA-treated cells were analyzed at the same gel. Surprisingly, in the raft fractions we could detect mainly a band migrating with M_r_ about 250,000 (Fig. 3C, left panel). It is not clear if this band corresponds to Octn2 oligomers or a stable complex with other protein(s), a phenomenon observed in case of some very hydrophobic proteins, even in SDS presence [43]. The amount of this high molecular weight species increased significantly after treatment with PMA, especially in fractions 4 and 5, a phenomenon reversed by Bis II.

### Raft Proteins Co-precipitate with Octn2

**Figure 4:**
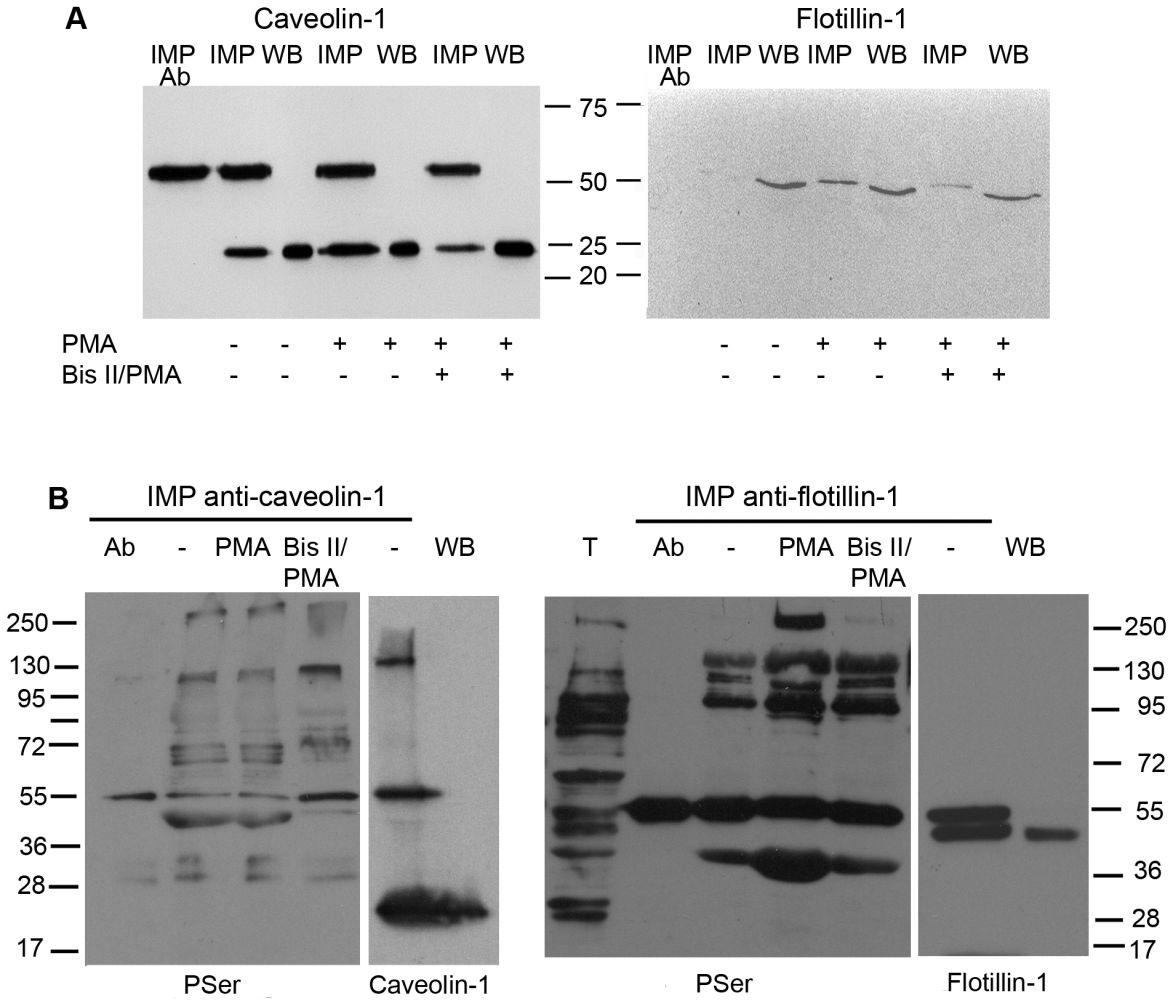
Interaction of Octn2 with raft proteins. (A) Samples subjected to immunoprecipitation (IMP) with anti-Octn2 antibody were either obtained from control astrocytes or after treatment with PMA and Bis II with PMA. Western blot analyses (WB) of total extracts were taken as controls. Ab – anti-Octn2 antibody taken for immunoprecipitation. (B) Verification of caveolin-1 and flotillin-1 phosphorylation. Cell extracts were subjected either to immunoprecipitation (IMP) with anti-caveolin-1 or anti-flotillin-1 antibodies and analysed for the presence of phosphoserine with the antibody recognizing PKC phosphorylation site. Ab, the corresponding antibody subjected to immunoprecipitation. As controls, the extracts were analysed for the electrophoretic mobility of raft proteins after immunoprecipitation and by Western blot. T – total protein extract (n = 4).

Detection of Octn2 in raft fractions led us to a more precise analysis of its possible interaction with raft proteins. As shown in Fig. 4A, activation of PKC and treatment with Bis II did not change total amount of rafts proteins. Caveolin-1 co-precipitates with Octn2 and its amount is almost doubled (193%) after PMA treatment. Interaction with flotillin-1 is hardly detectable under control conditions, anyhow, flotillin-1 can be detected after PMA treatment, what is significantly reversed by Bis II. Since we observed co-precipitation of phosphorylated proteins with Octn2, we wanted to verify if the raft proteins can be phosphorylated under applied experimental conditions. Immunoprecipitation of caveolin-1, as well as of flotillin-1 resulted in detection of several phosphorylated proteins, none of them, however, corresponded to caveolin-1 or flotillin-1 (Fig. 4B).

### Octn2 Contains Caveolin-1 Binding Domains

**Figure 5:**
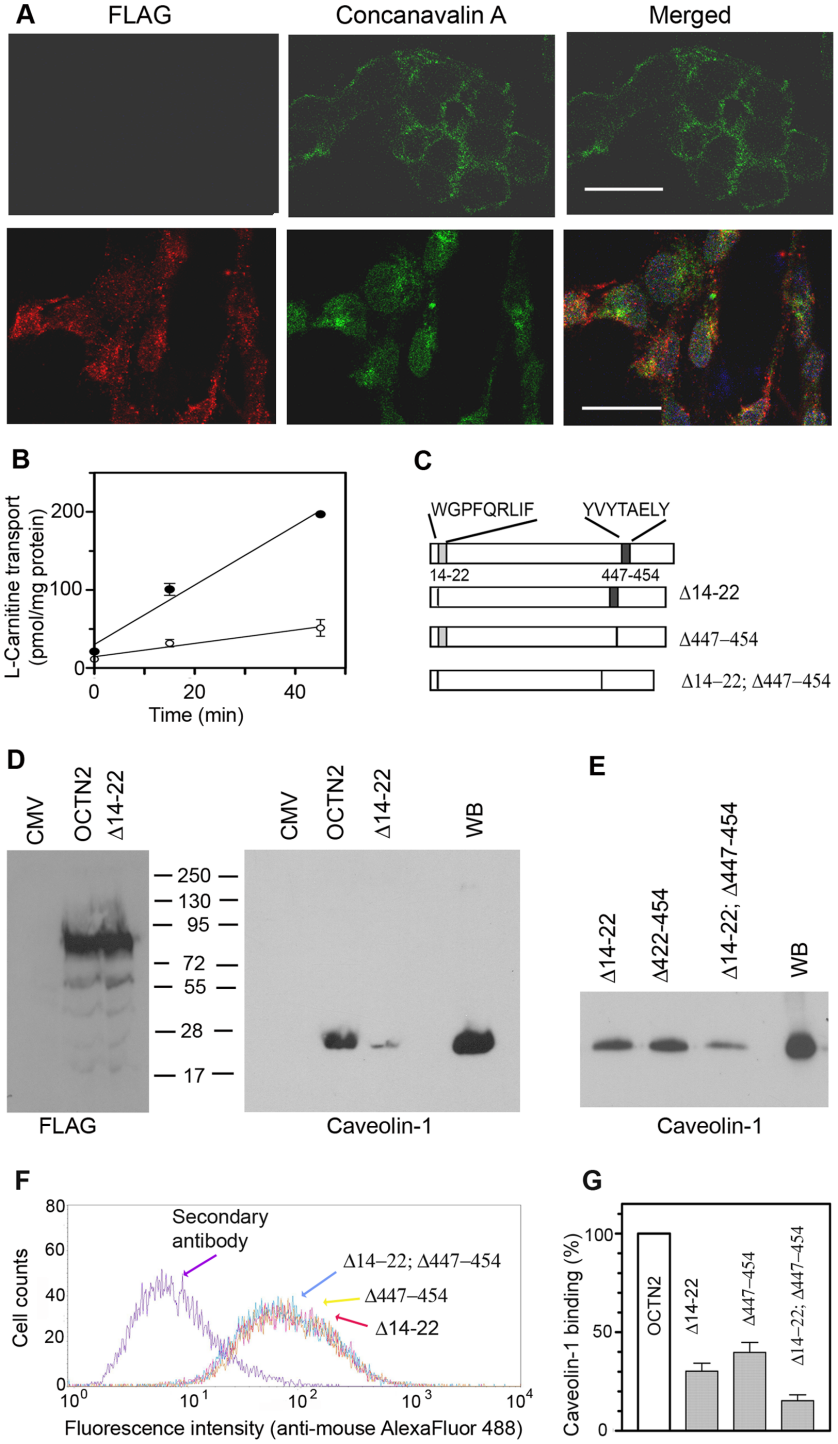
Verification of caveolin-1 binding to Octn2. Experiments were performed after overexpression of rOctn2 in HEK293 cells, as described in Materials and Methods. (A) Checking of Octn2 topology. HEK293 cells transfected with p3xFLAG-CMV14/OCTN2 were analysed with anti-FLAG antibodies or binding of concanavalin A in either non-permeabilized (upper panels) or permeabilized (lower panels) cells. Bar 25 µm. (B) Measurements of carnitine transport in HEK293 cells transfected with p3xFLAG-CMV14/OCTN2 (closed symbols) or with p3xFLAG-CMV14 alone (open symbols), the results are means ± SEM (n = 3). (C) Schematic presentation of rOctn2 after deletion of the potential caveolin-1 binding sites. (D) Immunoprecipitation (n = 6) of Octn2 wild type and Δ14–22 mutant (left panel). The right panel shows detection of caveolin-1 co-precipitating with Octn2 after transfection with p3xFLAG-CMV14/OCTN2 or p3xFLAG-CMV14/OCTN2-Δ14–22 using clones with an equal level of FLAG-tagged protein (left panel), n = 4. CMV, transfection with p3xFLAG-CMV14 vector alone. (E) Detection of caveolin-1 binding to Octn2 after selection of clones with equal FLAG levels (n = 4), as presented in (F). (G) Quantitative presentation of caveolin-1 co-precipitating with a wild type Octn2 and its deletion mutants normalized to the level of FLAG.

We wanted to verify if Octn2 can directly interact with raft proteins. There is no information on amino acid sequence responsible for flotillin-1 binding, anyhow, the motifs φxφxxxxφ, φxxφxxxxφ, φxxxxφxφ or φxxxxφxxφ, where φ corresponds to an aromatic amino acid residue (Trp, Tyr, Phe), while x can be any amino acid, can bind to caveolin scaffolding domain [44]. The proposed topology of OCTN2 assumes both N- and C-termini to be localized in the cytoplasm. We cloned cDNA coding rOctn2 with C-terminally fused 3xFLAG peptide and stably expressed it in HEK293 cells. As shown in Fig. 5A (upper panels), there is no reaction with anti-FLAG antibody when the cells were not permeabilized, although the cells were labeled with concanavalin A reacting with sugar moieties present at the outer side of plasma membrane. The anti-FLAG antibody detected the antigen after permeabilization (Fig. 5A, lower panels), what confirms proposed topology of Octn2. The over-expressed Octn2 is functionally active (Fig. 5B). Taking into account transporter topology, we further searched for caveolin-1 binding motifs at the intracellular loops of Octn2. The peptides corresponding to amino acids 14–22 and 447–554 were selected and mutants with a deletion of either site or a double-deletion were created (Fig. 5C). We obtained deletion mutant Δ14–22 with the same expression level as the wild type and binding of caveolin-1 by this protein was decreased by 70% (Fig. 5D, right panel). Deletion of amino acids 447–454 strongly affected Octn2 stability, since these mutants never reached the level of a FLAG-tagged wild type protein. Therefore, we selected the clones characterized by the same level of FLAG (Fig. 5F) and caveolin-1 binding was compared among three deletion mutants. As shown in Fig. 5E, the effect of deletion amino acids 422–454 on caveolin-1 binding is similar, as in case of the first deletion, while the double deletion decreased dramatically, although not completely, binding of this raft protein. Quantification of these observations, when normalized to FLAG level, indicates that both fragments can bind caveolin-1 and a simultaneous removal of both fragments leads to a further decrease (by 86.7±4.2%) of co-precipitating caveolin-1.

**Figure 6:**
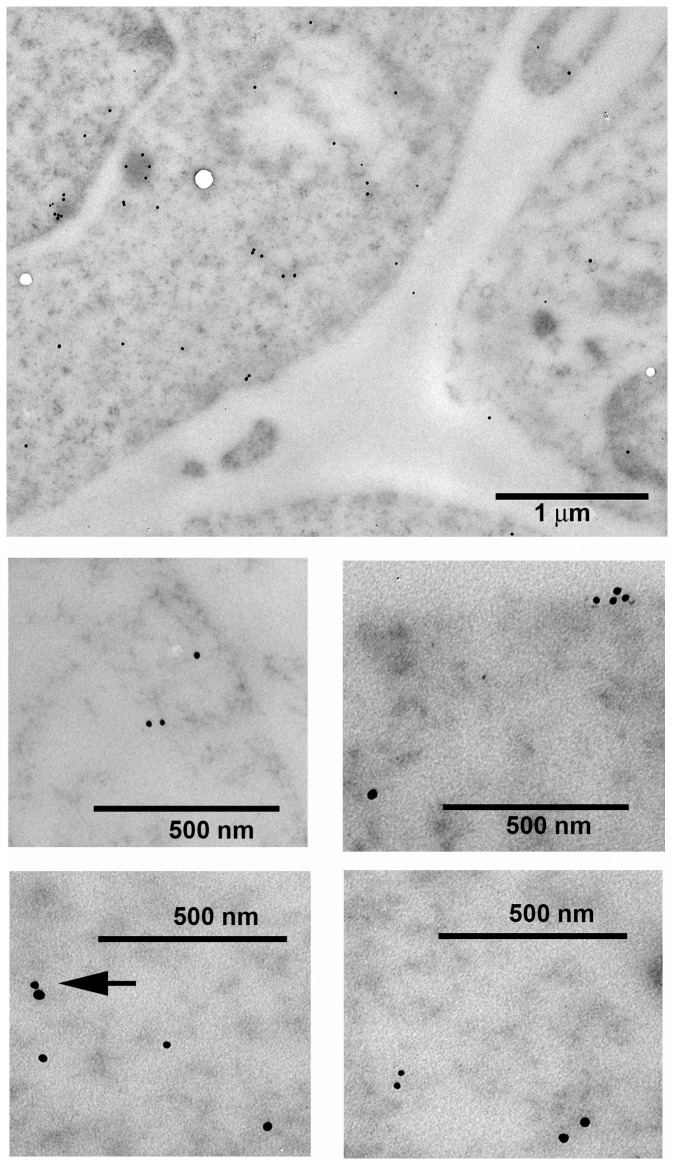
Localization of rOctn2 and OCTN2 in HEK293 cells. The cells were transfected with p3xFLAG-CMV14/OCTN2 and analysed by immunogold transmission electron microscopy as described in Materials and Methods. Presence of rOctn2 was detected with anti-FLAG antibodies (visualized with 10 nm colloidal gold particles), that of endogenous hOCTN2 with anti-OCTN2 antibody (with 15 nm colloidal gold particles). Arrow indicates heterodimer between endogenous OCTN2 and overexpressed Octn2.

Since the double deletion did not eliminate completely co-precipitation of mutated Octn2 with caveolin-1 and many transporters are known to form homooligomers, we wanted to verify if over-expressed Octn2 can form oligomers, in particular with the endogenous hOCTN2. Analysis with use of TEM confirmed Octn2 localization both in the plasma membrane and in the cytoplasm (Fig. 6), as detected previously in biotinylation studies. Presence of dimers and higher oligomers could be observed. Dimers detected simultaneously by anti-FLAG and anti-Octn2 antibodies were also visible (Fig. 6, arrow), what could explain residual binding of caveolin-1 by mutated proteins, after elimination of caveolin-1 binding domain.

### Octn2 Can Interact Directly with Caveolin-1 but Not with Flotillin-1

**Figure 7:**
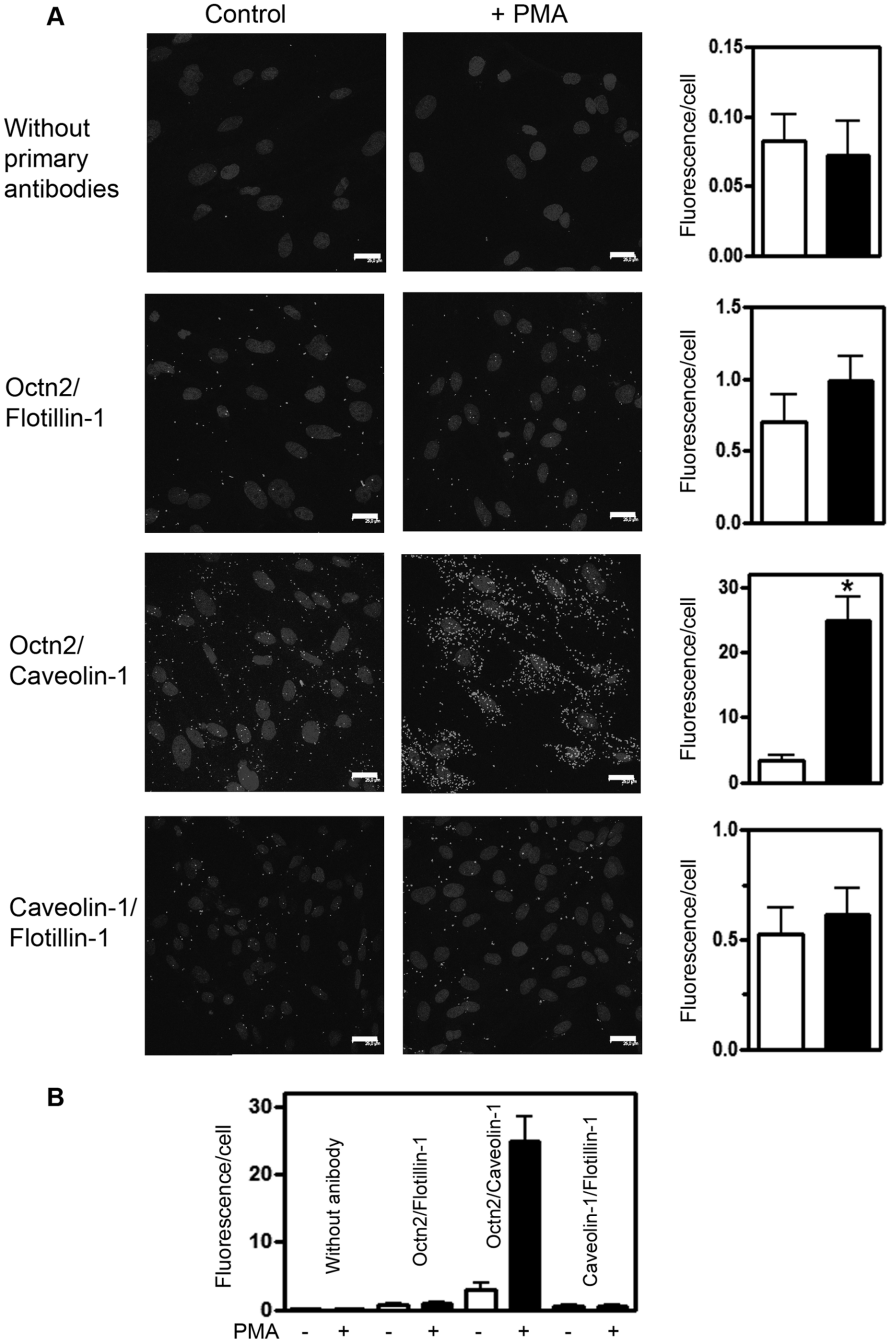
Verification of Octn2 binding to raft proteins in astrocytes. (A) The cells were treated as given in Materials and Methods either without (control) or with 200 nM PMA. Proximity ligation assays were performed with use of rabbit anti-Octn2 antibody and either mouse anti-flotillin-1 or mouse anti-caveolin-1 antibody with the samples not-treated with the primary antibodies as reference. A possibility of caveolin-1/flotillin-1 interaction was verified with use of rabbit anti-caveolin-1 and mouse anti-flotillin-1 antibodies. Quantification (shown in right panels) was performed by measurements of fluorescence referred to number of nuclei (n = 10), *p<0.001 towards control without PMA. (B) Summarized results of proximity ligation assays. Statistical significance towards all other experimental systems with p<0.001 was indicated only in case of Octn2/caveolin-1 interaction after PMA.

Co-precipitation of two proteins does not prove their direct interaction, therefore we used a proximity ligation assay which allows to detect interaction at the distance not exceeding 40 nm. As presented in Fig. 7A, fluorescence measured after PMA treatment was not different in case of Octn2/flotillin-1 antibodies, when compared with the cells treated with PMA. Moreover, it was not significantly different from the background measured without primary antibodies (Fig. 7B). There was a significant increase in fluorescence signal after PMA when anti-Octn2 and anti-caveolin-1 antibodies were used, what indicates a direct interaction between both proteins. Interestingly, there was no signal indicating a possibility of direct interaction between both raft proteins either under control conditions or after PMA (Fig. 7).

## Discussion

Octn2 present in rat astrocytes in majority is localized intracellulary ([6], present study). Such a phenomenon has been reported in case of many other plasma membrane transporters [32], [45]. It has to be noted that TEM analysis showed presence of Octn2 oligomers in plasma membrane and in cytoplasm [6]. Oligomeric structure was observed in case of several transporters coded by *SLC* superfamily of genes, as reported for hetero-trimers of glutamate transporters [46] and dimers of several proteins coded by *SLC6* family [47]–[49]. These observations confirm a conserved dimeric structure of the bacterial homologue of Na^+^-dependent transporters with 12 transmembrane domains [50]. Transporters oligomerization has been postulated to be important for exit from endoplasmic reticulum [51]. It seems quite probable that oligomers in plasma membrane could form the functional units of transporter, since the increase of Octn2 at cell surface after PMA is higher than increase of transporting activity.

Transporters trafficking was observed to be affected by PKC activation with phorbol esters [23], [29], [30], [33]. On the contrary to Rytting and Audus [52], who could not detect any effect of PMA on carnitine transport in choriocarcinoma trophoblasts, phorbol ester treatment correlated with an increased transport in astrocytes, although we could not detect any phosphorylation of Octn2. Anyhow, it has to be added that phosphorylation and trafficking were observed to be independent on each other in case of dopamine transporter [53].

The current study demonstrates a direct interaction between Octn2 and caveolin-1. Since caveolin-1 is known to form oligomers [54], it seems quite probable that the two fragments of Octn2 sequence, namely amino acids 14–22 and 447–454, bind independently to two caveolin-1 molecules, forming a multiprotein complex. Caveolin-1 can be phosphorylated by PKC on Ser^80^ [55], anyhow, we did not detect any phosphoserine in caveolin-1 in astrocytes and, although there were phosphorylated proteins co-precipitating with caveolin-1, their phosphorylation status was not changed by PMA. A direct interaction of Octn2 and caveolin-1 seems, however, very interesting, especially that caveolin-1 has been proposed not only to be a structural element in cholesterol-sphingolipid rich microdomains of plasma membrane, but to fulfill as well a role of chaperone protein inside the cell [56].

Our analysis of protein-protein interaction with proximity ligation assay excluded a direct interaction between caveolin-1 and flotillin-1 in astrocytes, does not exclude, however, their presence in a multiprotein complex and an indirect binding through other protein(s). Although flotillin-1 can be phosphorylated by PKC, what correlated with internalization of dopamine transporter [24], it is not phosphorylated in astrocytes. There are several proteins co-precipitating with either Octn2 or flotillin-1, in case of which PMA augmented serine phosphorylation and modification of these proteins could affect trafficking of Octn2, although their identity remains to be established. It is worth mentioning that formation of a ternary complex containing flotillin-1 was reported in case of glucose transporter GLUT4 delivery to plasma membrane [57]. Moreover, flotillins have been postulated to guide specific membrane proteins to the cell surface [58]. Therefore, we expect that such protein(s), whose phosphorylation is PKC-dependent, could bind to flotillin-1 and through this association facilitate indirect binding of flotillin-1 to caveolin-1, in consequence promoting transfer of Octn2 to plasma membrane and transporter localization in detergent-insoluble (raft) microdomains. Since this transfer is correlated with an increased activity of carnitine transport, these observations would imply that Octn2 localized in rafts is in its active form.

Oxidative metabolism of fatty acids in the brain was detected in astrocytes [16], therefore carnitine accumulation would be important for energy delivery. It should be emphasized that disorders of fatty acid oxidation lead not only to cardiomyopathy, muscle weakness, lipid accumulation in liver, but also to several neurological symptoms, as hypoketotic encephalopathy, Rey-like syndrome, seizures and mental retardation [10], [59] and some of them result directly from carnitine deficiency [60]. Carnitine is also essential for control of cellular CoA [61], the availability of which is necessary for synthesis of cholesterol in astrocytes and its subsequent delivery to neurons [18]. Formation of acylcarnitines in the brain, especially acetylcarnitine can affect neurotransmitter synthesis [62]. It has to be also added that, due to its ability to transport betaine [9], [63], a product of choline metabolism, Octn2 in astrocytes can play *in vivo* an important role in regulation of brain osmolarity. Octn2 regulation can be also important in understanding of secondary carnitine deficiency, known to be induced by several drugs, since OCTN2 is widely recognized as a drug transporter and among its substrates are β-lactam antibiotics, as well as oxaliplatin, varapamil, mildronate, valproate, imatinib and quinidine [9], [10], [64], [65]. A direct interaction between Octn2 and caveolin-1 seems to be a novel mechanism regulating the activity of this transporter.

## Conclusion

Our observations indicate that Octn2 can be regulated post-translationally by trafficking, a process dependent on formation of a multiprotein complex (containing caveolin-1 and flotillin-1) and regulated by PKC-dependent phosphorylation of other than Octn2 protein(s). Amino acids 14–22 and 447–454 of Octn2 sequence are involved in a direct interaction with caveolin-1. PKC activation directs this complex to rafts, leading in consequence to an increased transporting activity.
